# Does the kinorhynch have a hydrophobic body surface? Measurement of the wettability of a meiobenthic metazoan

**DOI:** 10.1098/rsos.160512

**Published:** 2016-10-19

**Authors:** Daisuke Ishii, Hiroshi Yamasaki, Ryosuke Uozumi, Euichi Hirose

**Affiliations:** 1 Department of Life Science and Applied Chemistry, Graduate School of Engineering, Nagoya Institute of Technology, Nagoya, Aichi 466-8555, Japan; 2 Department of Chemistry, Biology and Marine Science, Faculty of Science, University of the Ryukyus, Nishihara, Okinawa 903-0213, Japan; 3 Museum für Naturkunde Berlin, Leibniz Institute for Evolution and Biodiversity Science, Invalidenstrasse 43, 10115 Berlin, Germany

**Keywords:** contact angle, hydrophilic coat, hydrophobic cuticle, interstitial habitat, meiobenthos, wettability

## Abstract

The body surface of aquatic invertebrates is generally thought to be hydrophilic to prevent the attachment of air bubbles. In contrast, some interstitial invertebrates, such as kinorhynchs and some crustaceans, have a hydrophobic body surface: they are often trapped at the water surface when the sediment in which they reside is mixed with air and water. Here, we directly measured the wettability of the body surface of the kinorhynch *Echinoderes komatsui*, using a microscopic contact angle meter. The intact body surface of live specimens was not hydrophobic, but the anterior part was less hydrophilic. Furthermore, washing with seawater significantly decreased the wettability of the body surface, but a hydrophilic surface was recovered after a 1 h incubation in seawater. We believe that the hydrophobic cuticle of the kinorhynch has a hydrophilic coat that is readily exfoliated by disturbance. Ultrastructural observations supported the presence of a mucus-like coating on the cuticle. Regulation of wettability is crucial to survival in shallow, fluctuating habitats for microscopic organisms and may also contribute to expansion of the dispersal range of these animals.

## 1. Introduction

In aquatic environments, adhesion of air bubbles is a serious problem, particularly for small organisms; the bubbles may cause buoyancy problems and interfere with movement and water flow occlusion. In extreme cases, the organisms are trapped at the water surface. These problems are particularly crucial for epipelagic organisms, swimming larvae and those in benthic shallow or intertidal zones. For instance, ascidian larvae, when deprived of their vitelline coats and test cells, have a hydrophobic integument and are easily trapped at the water surface [1]. Theoretically, more bubbles adhere to hydrophobic surfaces, and aquatic organisms are thought to have hydrophilic bodies [2] and some appear to have elaborate hydrophilic coats on their bodies [1,3,4]. In contrast, some interstitial invertebrates have a hydrophobic body surface; Cloney & Hansson [1] noted that ‘many interstitial invertebrates that do not swim near the surface are hydrophobic (E. E. Ruppert, personal communication)’. By using their hydrophobic properties, some interstitial invertebrates can be collected on the water surface by the bubbling and blotting method [5–8]. However, some interstitial invertebrates live near the water surface and they run the risk of being trapped at the water surface.

Species of the phylum Kinorhyncha are marine, meiobenthic metazoans that occur in various marine sediments; e.g. mud, fine sand and coarse shell sand. They are distributed in the intertidal zone to the abyssal depths from the polar to the tropical regions. Almost all kinorhynchs can be extracted with the bubble and blot method [7,8], except some obligate intertidal species [9]. *Echinoderes komatsui* Yamasaki & Fujimoto [10] is a kinorhynch found in intertidal flats at river mouths where the water level, salinity and water temperature fluctuate daily owing to the ebb and flow of the tides; the surface of the tidal flat is often dry at low tide. Although this kinorhynch lives near the water surface, it can be collected by the bubbling and blotting method, indicating that it has a hydrophobic body. Does the kinorhynch actually have a hydrophobic body surface? The wettability for water, the degree of hydrophilicity/hydrophobicity, is usually evaluated with the contact angle of the water drop on the surface. However, kinorhynchs are too small (less than 0.1 mm in width) to measure wettability using a standard contact angle meter because of the difficulty of handling picolitres of water droplet that evaporates in a short time. Here, we used a microscopic contact angle meter equipped with high-speed camera to evaluate the wettability of *E*. *komatsui* by measuring two values in 30 pl water drops on the surface: contact angle (CA) and time it takes for a water drop to disappear (TIME). This is the first report on the measurement of wettability of meiobenthic invertebrates.

## 2. Material and methods

### 2.1. Animals

Sediment samples were taken by hand from an intertidal flat at a river mouth in Oura Bay, Okinawajima Island, Japan (26°33.35′ N, 128°2.57′ E). Some meiofaunal species, including *Echinoderes komatsui*, ostracods, tanaids, harpaticoids and nematodes, were extracted from the sediment by the bubbling and blotting method [7,8]. Subsequently, specimens of *E. komatsui* were sorted under a stereo microscope. The kinorhynchs used for wettability measurements were reared in 21‰ seawater for a few days, because the salinity of the interstitial water at the collection site was about 21‰. The specimens for electron microscopy were immediately fixed after the collection.

### 2.2. Measurement of wettability

Wettability was evaluated based on the contact angle of the water drop on the surface of the specimen and TIME owing to spreading on the body surface and evaporation. A drop spreads more readily on a hydrophilic surface and has a smaller contact angle and a shorter TIME than on a hydrophobic surface.

Each kinorhynch was gently transferred and placed on a glass slide, using a Pasteur pipette, and the excess water was removed with a piece of absorbent paper. All measured animals retracted their head into the body after the removal of the water. Then, a drop of distilled water (about 30 pl) was placed on the neck or trunk region of the body. The successive changes in drop shape were recorded at 50 fps, using a high-speed camera HAS-220 (Ditect, Japan) with a microscopic contact angle meter (MCA-3: Kyowa Interface Science, Japan). The measurement was performed for 13 animals without any prior treatments (intact specimens), for nine animals washed by pipetting in 21‰ seawater (washed specimens), and for two specimens incubated in 21‰ seawater for 1 h after the washing treatment (recovered specimens).

### 2.3. Microscopy

Specimens fixed in 2.5% glutaraldehyde, 0.1 M cacodylate and 0.45 M sucrose were post-fixed for 1.5 h in 1% osmium tetroxide and 0.1 M cacodylate following a brief rinse with buffer, and then dehydrated through ethanol. For scanning electron microscopy (SEM), the specimens were immersed in *t*-butanol, freeze-dried before sputter-coating with gold–palladium and examined using a JEM-6060LV (JEOL, Japan). For transmission electron microscopy, the specimens were cleared with *n*-butyl glycidyl ether and embedded in epoxy resin. Thick sections were stained with toluidine blue for light microscopy, and thin sections were stained with uranyl acetate and lead citrate and examined in a JEM-1011 (JEOL, Japan).

## 3. Results

**Figure 1.**
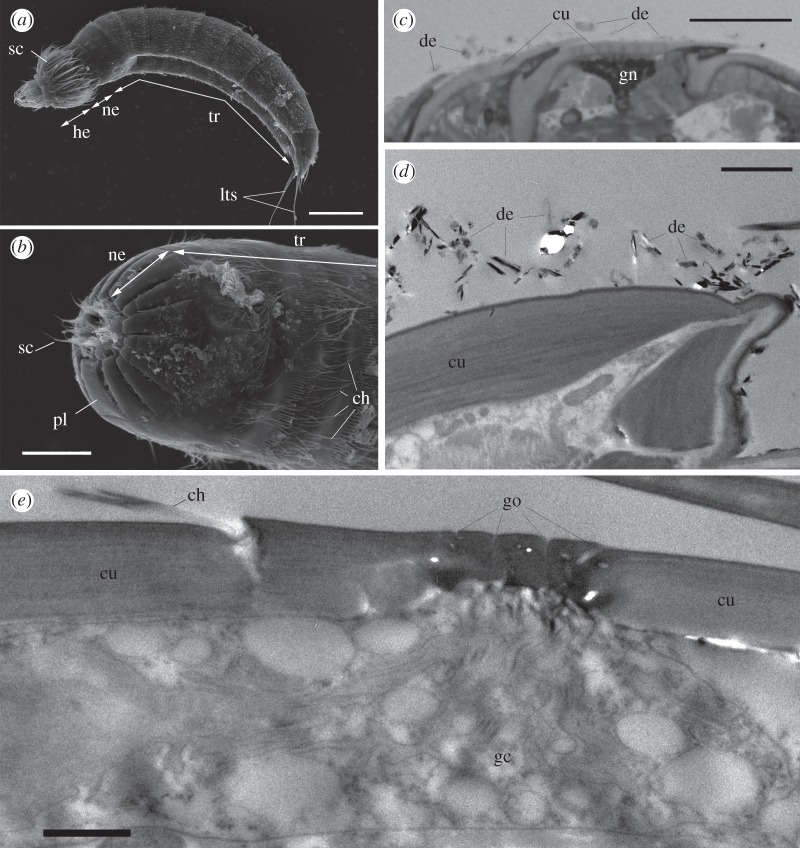
SEM (*a*,*b*), histological section (*c*), and TEM (*d*,*e*) images of *Echinoderes komatsui*. (*a*) Whole mount specimen. (*b*) Enlargement of neck and trunk segments 1–3. Head is retracted. (*c*) Sagittal section of the trunk covered with debris (de). Granular cell (gn) was found beneath the cuticle. (*d*) Trunk cuticle (cu) covered with debris (de). (*e*) Gland cell (gc) with glandular cell outlets (go) opening through the cuticular layer. ch, cuticular hair; he, head; lts, lateral terminal spine; ne, neck; pl, placid; sc, scalid; tr, trunk. Scale bars, 50 µm (*a*); 20 µm (*b*) and (*d*); 2 µm (*d*); 1 µm (*e*).

The animal body consisted of a head, neck and trunk (figure 1*a*); the head was mostly retracted in the body during the measurements. The neck comprised 16 placids with no hairs, whereas the trunk had 11 segments with numerous cuticular hairs (see [10]) (figure 1*b*). A pair of lateral terminal spines was present on the last (11th) segment. The cuticular surface was smooth, but the surface was often covered with debris that was probably trapped in the secreted mucus (figure 1*c*,*d*). In the histological section (figure 1*c*), granular cells were occasionally distributed beneath the cuticle. In TEM, gland cells were found beneath the trunk cuticle, and the glandular cell outlets opened on the cuticular surface (figure 1*e*).

**Figure 2.**
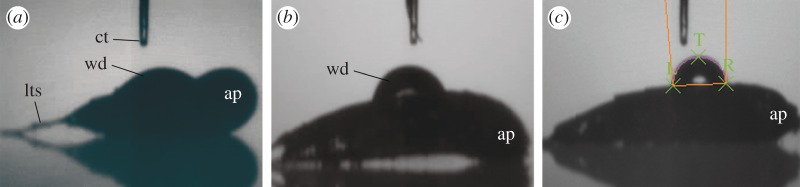
Captured images from the high-speed movies of the water drops on the middle part of *Echinoderes komatsui*. (*a*) Intact specimen. (*b*) Washed specimen. (*c*) Measurement of the contact angle. ap, anterior part; ct, capillary tip; lts, lateral terminal spine; wd, water drop.

Wettability was measured on three parts of the animal body: anterior (neck + anterior trunk), middle (middle trunk) and posterior parts (posterior trunk). Table 1 summarizes the averages of contact angle and TIME with standard deviations (s.d.) in intact and washed specimens; all measured values are given in electronic supplementary material, S1. Water drops on hydrophilic (intact) specimens have smaller contact angles and shorter disappearance times than those on hydrophobic (washed) specimens (figure 2 and electronic supplementary material, S2–S3). Based on the contact angles, the anterior part was less hydrophilic than the other parts in the intact specimens (Student–Newman–Keuls test: *p *< 0.05), whereas the variation in TIME among parts was not significant (ANOVA: *p *= 0.2922). In washed specimens, there was no variation in either contact angle or TIME among the three parts (ANOVA: CA, *p *= 0.7395; TIME, *p *= 0.3521). The washing treatment significantly decreased wettability of the middle part (*t*-test: CA, *p *< 0.01; TIME, *p *< 0.01) and posterior part (*t*-test: CA, *p *< 0.05; TIME, *p *< 0.05), whereas neither contact angle nor TIME differed significantly on the anterior part (*t*-test: CA, *p *= 0.2818; TIME, *p *= 0.1269). After a 1 h incubation in seawater following the washing treatment, wettability was recovered on the middle part (*n*, 2; CA 23.6° ± 1.83; TIME, 480 ms ± 396.0) and posterior part (*n*, 2; CA 39.7° ± 3.11; TIME, 930 ms ± 636.4), whereas the anterior part was still hydrophobic (*n*, 1; CA, 151°; TIME, 4060 ms).

**Table 1.** Measurements of the wettability in intact and washed specimens.

	intact			washed			
part of the body	*n*	average	s.d.	*n*	average	s.d.	difference^a^
	contact angle (°)						
anterior	11	77.4	18.1	8	87.4	20.9	*p *> 0.05
middle	13	59.0	15.7	9	80.9	12.9	*p *<* *0.01
posterior	10	60.1	21.1	9	84.5	17.4	*p *<* *0.05
	TIME (msec)^b^						
anterior	11	1316	852.8	8	1890	630.9	*p *>* *0.05
middle	13	935	860.2	9	2214	892.0	*p *<* *0.01
posterior	10	780	635.3	9	1651	864.1	*p *<* *0.05

^a^Unpaired *t*-test.

^b^The time it takes for a water drop to disappear.

## 4. Discussion

In intact specimens of *E*. *komatsui*, the middle and posterior parts had hydrophilic surfaces (about 60° [CA], < 1 s [TIME]), whereas the anterior part (neck + anterior trunk) was less hydrophilic (nearly 80° [CA], >1 s [TIME]). Wettability varied among the specimens, probably because of the extent of damage during handling of the specimens. Wettability on the middle and posterior parts significantly decreased after the washing treatment (about 80° [CA], > 1 s [TIME]), indicating that a hydrophilic coat covers the hydrophobic trunk *in situ* and this was removed by pipetting. Because the difference in the wettability on the anterior part was not significant between intact and washed specimens, the anterior part is not or is rarely covered by a hydrophilic coat. Following a 1 h incubation in seawater, the middle and posterior parts recovered their wettability, but the anterior part remained hydrophobic. Ultrastructural observations suggest that the hydrophilic coat is a mucus layer secreted from gland cells via glandular outlets opening on the cuticular surface of the trunk.

Yamasaki & Fujimoto [10] observed that *E*. *komatsui* has two types of glandular cell outlets that open on each trunk segment; the outlets shown in figure 1*e* correspond to outlet type 1, characterized by small, multiple openings. The granular cell in figure 1*c* may be also a type of glandular cell, whereas the presence of glandular cell outlets was not confirmed in the present observation. Several types of gland cell have been described in many kinorhynchs, and some studies have reported a mucus layer on the trunk surface [9,11]. The trunk segments of *E*. *komatsui* are densely covered with cuticular hairs, and these hairs appear to prevent the loss of the hydrophilic coat. Dense hairs covering the trunk segments are shown in many *Echinoderes* species [9,10,12], and the hairs may have a similar function at least in intertidal and shallow-water species that usually face a threat of attaching air bubbles on their body surface. Among the parts of the body, the anterior part was less hydrophilic than the middle and posterior parts, and recovery of wettability in this part did not occur during the 1 h incubation period after washing treatment. Kinorhynchs always repeat retraction and projection of the head for locomotion, and the mucus layer on the anterior part would be more easily exfoliated than the other parts owing to the active movement of the head.

**Figure 3.**
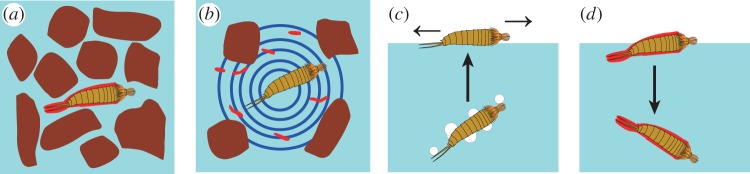
Possible process of the change of wettability on kinorhynch body and long-distance dispersal by drifting on the sea surface. (*a*) Kinorhynch body has a hydrophilic coat (red) in interstitial habitat in intertidal flat. (*b*) Disturbance of the habitat causes exfoliation of the hydrophilic coat. (*c*) Attachment of bubbles on the hydrophobic cuticle. Kinorhynchs are trapped on the sea surface and drift away. (*d*) Secretion of hydrophilic coat and re-settlement on the sea floor.

Unlike many aquatic invertebrates, kinorhynchs are thought to have a hydrophobic body, because they are trapped on the water surface by the bubbling and blotting method. This study, however, shows that the body surface of *E. komatsui* is hydrophobic, but that they have a hydrophilic trunk, probably owing to a hydrophilic coat. We believe that bubbles rarely adhere to the body of *E. komatsui* in their natural habitat. Moreover, they may expand their dispersal ability by controlling the wettability of their body surface. In the habitat of *E*. *komatsui*, breaking waves and fluctuations in water temperature produce tiny bubbles in the water. Under normal conditions, bubbles rarely adhere to a body with a hydrophilic coat. When the hydrophilic layer is exfoliated by disturbance under stormy conditions, the dense cuticular hairs allow bubbles to more readily adhere to the body surface; the bubbles on the hydrophobic, hairy surface are thought to get into the gap among the hairs and the cuticle and be less detachable than the bubbles on a smooth surface. Consequently, the bubbles become attached to kinorhynchs, allowing them to float on the water surface, where they drift over the sea, resulting in long-distance dispersal. Later, secretion of hydrophilic mucus may restore wettability and the drifting animals settle on the sea floor again (figure 3).

The mechanisms of kinorhynch dispersal have been rarely discussed: some intertidal species of *Echinoderes* are distributed along oceanic currents [13], bottom currents may transport *Campyloderes* sp. inhabiting mainly the deep-sea floor [14], and *Echinoderes ohtsukai* may expand its distribution range associated with the oyster culture [15]. The regulation of a hydrophilic/hydrophobic body surface with the presence/absence of a hydrophilic mucus layer could help long-distance dispersal of kinorhynchs by drifting, especially in intertidal and shallow-water *Echinoderes* such as *E. komatsui*. It will be interesting to investigate the wettability of other kinorhynchs inhabiting subtidal and deeper zones and examine whether they have an ability to regulate the wettability in a future study. In addition, some other interstitial organisms are trapped by the bubbling and blotting method, such as ostracods, tanaids, harpaticoids and nematodes; they would have a hydrophobic body surface as kinorhynchs have, and they may possess similar or other mechanisms controlling the wettability of their body surface. It is also possible that the regulation of the wettability may involve other functions that are valuable to meiobenthic organisms.

## Supplementary Material

EMS1 Measurement of the wettabily of a kinorhynch body: Measured values of each specimen and each condition

## Data accessibility

The dataset supporting this article is accessible in the electronic supplementary material.
